# How to tackle antimalarial resistance?

**DOI:** 10.15252/emmm.201607295

**Published:** 2016-12-27

**Authors:** Didier Leroy

**Affiliations:** ^1^ Medicines for Malaria VentureGenevaSwitzerland

**Keywords:** Microbiology, Virology & Host Pathogen Interaction, Pharmacology & Drug Discovery

## Abstract

In the context of drug resistance, malaria is no exception. Even the latest artemisinins have started to lose their full efficacy in patients. Didier Leroy tells us about the strategies adopted by Medicines for Malaria Venture to reduce this terrible culprit.

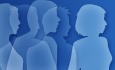

Life has a prodigious capacity to overcome death, and this is exactly emblematic of drug resistance, one of the biggest threats for health systems, clinicians, researchers and patients. Several decades of intense and innovative drug discovery and development were not able to prevent the loss of medicines owing to resistance among viruses, bacteria and parasites. The common denominator to drug resistance is the ability of the targeted microorganisms to select the very few of them that can overcome the selective pressure and grow, often as a result of mutations occurring in genes coding for either drug targets, drug transporters and efflux pumps, or drug degradation systems. In the context of malaria, even the latest generation of drugs, such as the artemisinins that were not supposed to act through a unique target and thus able to resist resistance mechanisms, has started to lose their full efficacy in patients.

In wild‐type parasites, the endoperoxide bond of artemisinin and its derivatives is cleaved in the presence of haem‐Fe^2+^ liberated from proteolyzed haemoglobin. Two processes have been described; the first generates oxygen radicals, which, via hydrogen atom abstraction or β‐scission processes, lead to carbon‐based radicals that can form covalent bonds with proteins and lipids in close vicinity. The second process involves a two‐electron heterolytic cleavage, in which the peroxide's oxidation potential causes death of the parasite (Haynes *et al*, 2013). The resulting alkylations or oxidative stress become extremely toxic to the parasite and inactivate vital functions. The cleavage of endoperoxides occurs mainly in the parasite's trophozoite blood stage where haemoglobin proteolysis takes place, and in very young ring stages, which makes artemisinins and all endoperoxides fast‐acting molecules.

Most of the endoperoxides, with the exception of the ozonide OZ439, are short‐lasting molecules. Once activated, artemisinins and endoperoxides kill parasites very fast, making them extremely powerful antimalarials. However, as most of these molecules have short human half‐lives, they need to be combined with long‐lasting drugs. Since the mid‐1980s, artemisinins and artemisinin‐derived molecules have been used in combination therapy with partner drugs so as to avoid the development and spread of drug resistance. The rationale was that the probability that two rare mutations that confer drug resistance occurring simultaneously even in billions of parasite genomes during an infection is close to zero compared to a single mutational event—provided that both drugs are used long enough to protect each other at high parasitaemia.

However, despite all these efforts, clinical resistance to artemisinin was first demonstrated in 2009 (Dondorp *et al*, 2009). More recently, artemisinin combination therapies (ACTs) failed in patients in Cambodia owing to drug resistance. This atypical resistance to artemisinins—called partial resistance by WHO—manifested first in the clinic, but drug potency in standard *in vitro* growth inhibition assays remained intact. The mechanism of resistance is caused by mutations in the gene encoding Kelch13, a protein domain that is involved in sensing intracellular oxidative stress following reactions of artemisinins and potentially other endoperoxides. Today, one of the most probable hypotheses to explain the molecular mechanism of resistance to artemisinin is based on the possibility that the function of Kelch13 is similar to mammalian Keap1, a protein that binds to a transcription factor and limits the antioxidant response. Mutated Kelch13 would no longer bind this transcription factor and stress‐mediated cell survival could take precedence, thereby leading to resistance (reviewed in Tilley *et al*, 2016).

Of additional concern is the recent finding that the partner drug piperaquine shows diminished efficacy correlating with an increase of the copy number of the *plasmepsin 2* and *plasmepsin 3* genes and a decreased copy number of *Pfmdr1* (*Plasmodium falciparum* multi‐drug resistance 1) in *Plasmodium* clinical isolates from Cambodia (Amato *et al*, 2016; Witkowski *et al*, 2016). Plasmepsins are proteases that digest haemoglobin in the parasite's food vacuole. The overexpression of plasmepsins could counteract the action of piperaquine, a situation that would be enhanced by a down regulation of PfMDR1 and the subsequent decreased incorporation of piperaquine in the food vacuole.

Facing an increasingly dramatic situation for malaria patients, organizations such as Medicines for Malaria Venture (MMV), in collaboration with its academic and industrial partners, WHO, donors and key opinion leaders, agreed to develop a strategy to protect the entire R&D pipeline of antimalarials against resistance. First, it was decided in 2009 to exclude new short‐lasting molecules that, like Artemisinin, contain an endoperoxide moiety and share the same mode of action. Concomitantly, it became crucial to develop new tools and approaches for anticipating and measuring the risk of drug resistance and its spread in the field.

A panel of well‐known and well‐characterized mutated strains of *Plasmodium* was assembled, which is now used routinely in parallel with field isolates from Africa and South‐East Asia to assess their level of sensitivity to any new lead molecule in discovery programmes. Molecules that show no signs of cross‐resistance are then used at suboptimal concentrations to induce resistance in cultured parasites at different levels of inoculum. The inoculum at which parasites survive the drug (10^5^–10^9^ parasites) defines the minimum inoculum for resistance or MIR: the lower it is, the higher the probability that *in vitro* drug resistance occurs. The second key parameter is the sensitivity of the surviving parasites to the compound evaluated via dose–response curves and the resulting loss of potency (increase of IC50) compared to a sensitive parasite strain. In antimalarial drug development, a drop of up to 10‐fold of *in vitro* potency is a signal that needs consideration, yet it is not a showstopper for the candidate molecule. Importantly, the evaluation of the fitness cost of each drug‐resistant microorganism—in other words the price, in terms of growth rate, to pay for survival in the presence of the drug over the drug‐sensitive organism—is the topic of another study. Combining these three parameters allows a fairly good assessment of the risk of drug resistance for each new lead molecule in the laboratory and helps to flag risk factors for future investigation, even if we cannot fully predict yet when and how fast drug resistance could appear and spread in the field.

During the last decade, MMV and its partners have developed seventeen candidates of which three are in Phase I (SJ733, P218 and MMV048), two are in Phase II (KAE609 and DSM265), and two have either completed or are part of ongoing Phase IIb studies (KAF156‐LUM, OZ439‐PPQ (Macintyre *et al*, 2016) and OZ439‐FQ). When testing combinations, MMV and WHO have integrated drug resistance research tools and strategies in the protocol of their clinical studies. Briefly, the goal, in this respect, is to verify the three main aspects of drug resistance by correlating the clinical decline of efficacy observed in patients with genotyping the same parasites isolated from the patients to highlight mutations in resistance markers and/or drug target genes and the loss of efficacy of each individual component of the combination measured *in vitro* on the same parasites.

A striking question is whether some chemical structures or features could be more prone to the development of drug resistance. Given the mechanism by which overexpression of efflux pumps such as P‐glycoprotein 1/MDR1 expulses xenobiotic molecules, one could hypothesize that this generic mode of resistance would not depend very much on the structure of the drug. In practice, some molecules have been shown to be “resistance proof”, suggesting that neither generic mechanisms of resistance, such as efflux pumps or drug degradation systems like glutathione‐S‐transferase, nor specific mutations in drug target genes seemed to have played a role. Understanding why a specific chemistry is more capable than another to select parasites with overexpressed and/or mutated genes is a key question for drug discovery and development in all therapeutic areas.

A recent study identified a hot spot for mutation in the mTOR kinase gene along with a chemical that targets mTOR, yet refractory to drug resistance induction. This could be the basis for predicting and designing a new generation of antitumor drugs that might resist drug resistance (Wu *et al*, 2015). In tuberculosis, mutation hot spots in genes encoding for RNA polymerase B (the target of rifampicin) or gyrase A (the target of fluoroquinolones) are associated to specific geographical localizations. This again shows that not all the drugs, patients and regions in the world are equivalent with respect to drug resistance. The structural aspects of chromatin and its accessibility to the molecular machinery that repairs mutations support the notion that the localization of drug target genes in the DNA could therefore be a key determinant for accumulating more (or less) mutations compared to other loci (Makova & Hardison, 2015). Consequently, DNA topological studies could predict potential risks for drug resistance.

In conclusion, antimalarial drug resistance is now better understood *in vitro*. The predictability of drug resistance happening in a controlled human malaria infection model or in the field, in patients, can consequently be assessed provided a resistance marker is available. Reinforcing such preclinical and clinical testing of drug candidates for resistance risk can therefore help to expand the usefulness of new drugs be they antimalarials or antibacterials.
